# The potential association between oral lichen planus and vaccination against hepatitis B. A real-world study analyzing data from approximately 200,000 patients

**DOI:** 10.1186/s12903-025-06565-3

**Published:** 2025-08-26

**Authors:** Borislav Todorov, Max Heiland, Susanne Nahles, Robert Preissner, Saskia Preissner, Moritz Hertel

**Affiliations:** 1 Department Oral and Maxillofacial Surgery, Charité– Universitätsmedizin Berlin, Augustenburger Platz 1, Berlin, 13353 Germany; 2 Institute of Physiology and Science-IT, Charité– Universitätsmedizin Berlin, Philippstrasse 12, Berlin, 10115 Germany

**Keywords:** Hepatitis B vaccines, Oral lichen planus, Lichenoid eruptions, Mouth, Vaccination

## Abstract

**Background:**

Oral lichen planus (OLP) and oral lichenoid lesions (OLL) are immune-mediated conditions that may occur in response to medications or vaccines. Although hepatitis B vaccination is widely considered safe, isolated case reports have suggested a possible link to OLP/OLL. However, large-scale epidemiologic data are lacking.

**Methods:**

A matched-cohort study was conducted using real-world data from the TriNetX database. The initial dataset included 112,444 patients who received hepatitis B vaccination and 7,002,541 unvaccinated individuals. After one-to-one propensity score matching, each cohort comprised 112,444 subjects. The primary outcome was the onset of OLP/OLL within 30 days post-index, identified via ICD-10 codes and confirmed histologically.

**Results:**

OLP/OLL occurred in 423 vaccinated patients (0.736%) and in 316 unvaccinated individuals (0.281%), with a significant group difference (*p* < 0.001, Log-Rank test). The risk ratio was 1.400 (95% CI: 1.210–1.619), and the odds ratio was 1.401 (95% CI: 1.211–1.622).

**Conclusion:**

This study demonstrates a statistically significant but modestly increased risk of OLP/OLL following hepatitis B vaccination. Given the small effect size and observational design, the findings should be interpreted as hypothesis-generating. Further prospective studies are warranted.

## Introduction

Hepatitis B is a viral infection caused by the hepatitis B virus (HBV), which can lead to chronic liver disease, cirrhosis, and hepatocellular carcinoma. Vaccination with recombinant hepatitis B surface antigen (HBsAg) is widely used to induce long-term immunity and prevent HBV infection [1]. Although the vaccine is generally considered safe, there have been isolated reports of adverse effects, including immunologically mediated skin and mucosal reactions such as lichen planus (LP) [2].

LP is an autoimmune disease characterized by inflammatory lesions of the skin and mucous membranes [3]. When localized in the oral cavity, the condition is referred to as oral lichen planus (OLP). In contrast, oral lichenoid lesions (OLL) represent delayed-type hypersensitivity reactions to external agents such as dental materials or systemic medications, including NSAIDs, beta-blockers, and ACE inhibitors [4, 5]. Despite different etiologies, OLP and OLL share substantial clinical and histopathological overlap, which makes reliable differentiation difficult in routine practice [5]. Accordingly, both conditions are jointly referred to as OLP/OLL throughout this study.

OLP/OLL typically present as reticular white striae, plaques, or erosive and atrophic lesions. Histologically, the key feature is a T-cell-mediated immune response targeting basal keratinocytes, resulting in apoptotic cells known as Civatte bodies [6].

Although LP and OLL have occasionally been reported following vaccination, including cases involving the hepatitis B vaccine, the existing evidence largely relies on case reports and small case series [7–9]. Due to the lack of large-scale epidemiologic data, the relationship between hepatitis B vaccination and the onset of OLP/OLL remains unclear. Therefore, the aim of this study was to investigate this potential association using real-world data from a large international patient database and to assess the relative risk of OLP/OLL in vaccinated versus unvaccinated individuals.

## Methods

### Data source and study design

The study design followed a previously established protocol for real-world analyses of vaccine-related adverse events [10]. This retrospective cohort study was conducted using anonymized patient data from the TriNetX Global Health Research Network (Cambridge, MA, USA), which aggregates electronic medical records from over 120 healthcare organizations across 19 countries. The study design followed a previously established protocol for real-world analyses of vaccine-related adverse events. To ensure computational feasibility, the dataset was limited to patients with a body mass index between 19.00 and 30.00 kg/m². Two cohorts were formed based on exposure to the hepatitis B vaccine: individuals receiving the vaccine at the time of an inpatient encounter (cohort I), and those without any documented hepatitis B vaccination (cohort II). Patients with a pre-existing diagnosis of lichen planus were excluded. Only records with complete follow-up data were considered. To account for potential confounding due to oral manifestations of chronic graft-versus-host disease (cGVHD), we conducted an additional screening of both cohorts for diagnostic codes indicative of prior allogeneic hematopoietic stem cell transplantation. No such cases were identified in either cohort, allowing us to exclude cGVHD as a relevant confounder in this analysis.

### Matching strategy

To reduce confounding and approximate randomization, a one-to-one propensity score matching (PSM) approach was applied using a greedy nearest neighbor algorithm with a caliper width of 0.25 standard deviations. Matching variables included age, gender, recent SARS-CoV-2 infection, COVID-19 vaccination within 120 days before or 30 days after the index event, presence of chronic viral hepatitis (which encompasses hepatitis C virus (HCV) infection as a known potential confounder), and use of medications previously associated with oral lichenoid reactions (ACE inhibitors, beta blockers, NSAIDs).

### Outcome definition

The primary outcome was the occurrence of OLP or OLL, identified using the following ICD-10 codes: L43, L44.2, and K13.7. Diagnoses were based on histopathological confirmation. In the TriNetX database, diagnoses of OLP and OLL are based on histopathological confirmation. It is important to note that histopathological evaluation is typically only performed in the presence of clinical signs suggestive of OLP/OLL, such as symptomatic mucosal lesions. Therefore, it can be assumed that all included cases also exhibited corresponding clinical manifestations. The index event was defined as the inpatient encounter date with hepatitis B vaccination (cohort I) or inpatient encounter alone (cohort II). A 30-day follow-up window was applied post-index to monitor the onset of the outcome.

### Statistical analysis

Following PSM, absolute risks, risk ratios (RR), odds ratios (OR), and their respective 95% confidence intervals (CI) were calculated. Time-to-event data were analyzed using the Log-Rank test. A p-value ≤ 0.05 was considered statistically significant. Statistical processing was performed directly within the TriNetX platform.

## Results

### Cohort characteristics

**Fig. 1:**
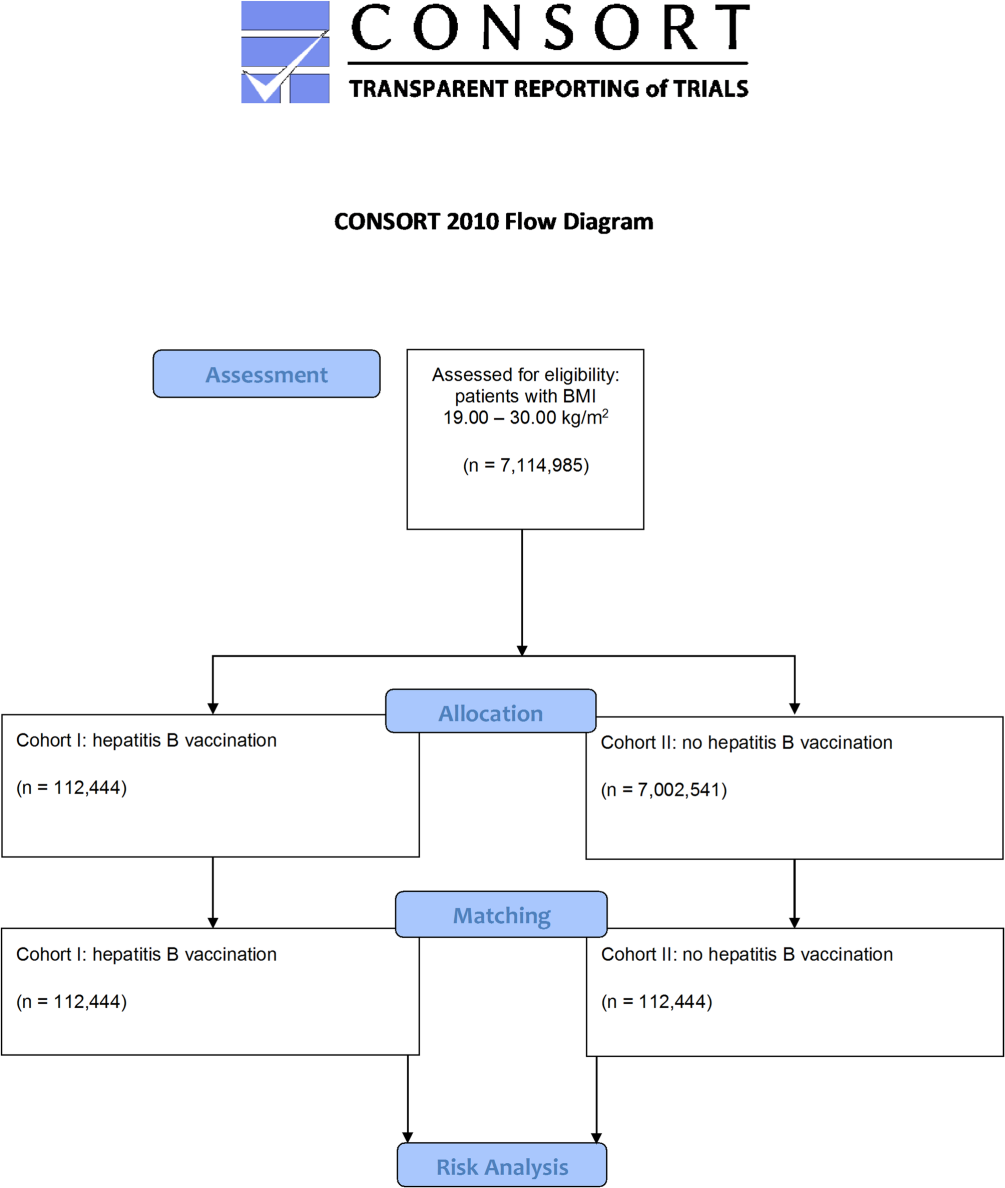
Modified Consolidated Standard of Reporting Trials (CONSORT) flow chart

**Fig. 2:**
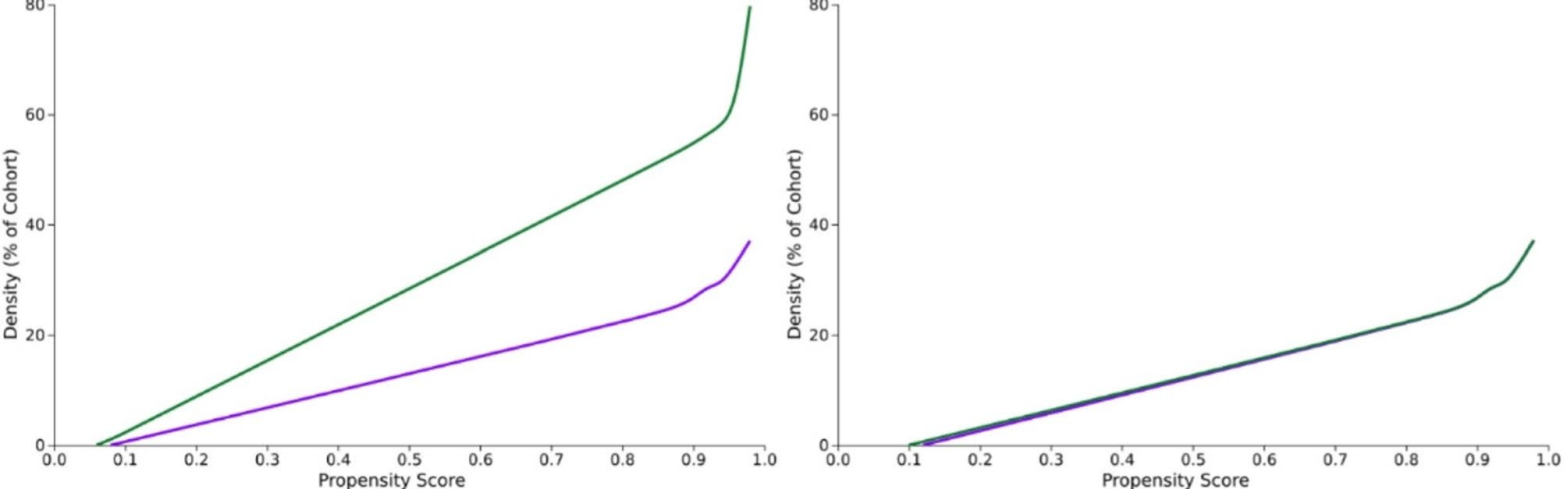
**A and B** Density of the cohorts I and II before and after propensity score matching for age, gender distribution, severe acute respiratory syndrome coronavirus (SARS-CoV)-2 infection, vaccination against coronavirus disease (COVID)-19, chronic viral hepatitis, use of angiotensin conversion enzyme (ACE) inhibitors, beta blockers, and non-steroidal anti-inflammatory drugs (NSAIDs). Cohort I (purple): patients who were vaccinated against hepatitis B and cohort II (green): subjects who did not receive hepatitis B vaccine

The access date was June 21, 2024. 92 HCOs provided medical records. Before matching, cohorts I and II accounted for 112,444 and 7,002,541 patients, respectively. After one-to-one propensity score matching, 112,444 subjects (53.4% female and 46.6% male; mean age 38.5 ± 22.2 years) remained per cohort. The data extraction process is displayed in Fig. 1. The characteristics of the cohorts before and after matching are shown in Table 1. The density of the cohorts before and after matching can be found in Fig. 2A and B.

The diagram illustrates the data extraction process from the TriNetX database. The total cohort size of *n* = 7,114,985 consisted of 112,444 individuals who received hepatitis B vaccine at inpatient encounter and 7,002,541 subjects who were not vaccinated. Vaccines used are listed in Table 2. The database was accessed on June 21, 2024. 

**Table 1:** Characteristics of cohorts I (vaccination against hepatitis B at inpatient encounter) and II (no vaccination against hepatitis B) before and after propensity score matching for age, gender distribution, severe acute respiratory syndrome coronavirus (SARS-CoV)-2 infection, vaccination against coronavirus disease (COVID)-19, chronic viral hepatitis, use of angiotensin conversion enzyme (ACE) inhibitors, beta blockers, and non-steroidal anti-inflammatory drugs (NSAIDs). SD = standard deviation, std. diff. = standardized difference

Cohort I (*n* = 112,444) and cohort II (*n* = 7,002,541) before propensity score matching									
	Demographics								
		Cohort			Mean ± SD	Patients	% of cohort	p-value	Std diff.
		I		Age	38.5 +/- 22.2	112,444		< 0.001	0.587
		II			51.7 +/- 22.5	6,902,750			
		I		Females		60,077	53.4	< 0.001	0.019
		II				3,622,622	52.5		
		I		Males		52,367	46.6		
		II				3,379,919	47.5		
	**Medical conditions**								
		I		COVID-19		6,612	5.9	< 0.001	0.216
		II				121,484	1.8		
		I		Chronic viral hepatitis		7,651	6.8	< 0.001	0.321
		II				52,746	0.8		
	**Medication**								
		I		ACE inhibitors		19,598	17.4	< 0.001	0.268
		II				586,006	8.5		
		I		Beta blockers		28,345	25.2	< 0.001	0.261
		II				1,023,877	14.8		
		I		NSAIDs		49,950	44.4	< 0.001	0.729
		II				921,935	13.4		
		I		SARS-CoV-2 vaccine		11,887	10.6	< 0.00	0.329
		II				174,536	2.5	1	
**Cohort I (n = 112**,**444) and cohort II (n = 112**,**444) after propensity score matching**									
	**Demographics**								
		I		Age	38.5 +/- 22.2	112,444		0.691	0.002
		II			38.6 +/- 22.2	112,444			
		I		Females		60,062	53.4	0.926	< 0.001
		II				60,040	53.4		
		I		Males		52,382	46.6		
		II				52,404	46.6		
	**Medical conditions**								
		I II		COVID-19		6,588	5.9	0.505	0.003
						6,514	5.8		
		I II		Chronic viral hepatitis		7,612	6.8	0.900	0.001
						7,627	6.8		
	**Medication**								
		I		ACE inhibitors		19,569	17.4	0.710	0.002
		II				19,636	17.5		
		I		Beta blockers		28,308	25.2	0.819	0.001
		II				28,355	25.2		
		I		NSAIDs		49,912	44.4	0.832	0.001
		II				49,962	44.4		
		I II		SARS-CoV-2 vaccine		11,846	10.5	0.967	< 0.001
						11,852	10.5		

### Risk analysis

Among cohorts, I and II, 423 and 316 individuals were diagnosed with OLP/OLL after the index event within the time window. The corresponding risks of 0.376% and 0.281% were significantly different (*p*<0.001; Log-Rank test). RR and OR were 1.400 (CI95%: 1.210–1.619) and 1.401 (CI95%: 1.211–1.622). The results of the risk analysis are summarized in Table 3.

**Table 2:** Distribution of hepatitis B vaccine brands and dosage strengths within cohort I

**(a) Vaccine brands administered**	
**Vaccine brand**	**Proportion (%)**
Pediarix	51.49%
Engerix-B	12.16%
Recombivax	8.48%
Twinrix	7.07%
Generic	6.99%
Vaxelis	6.61%
Heplisav-B	6.60%
Comvac	0.46%
Prehevbrio	0.12%
**(b) Vaccine dosage strengths**	
**Dosage strength (mg/mL)**	**Proportion (%)**
0.005	1.70%
0.01	13.61%
0.02	75.92%
0.04	8.77%

## Discussion

Lichen planus (LP) has been linked to both chronic liver disease and, in rare cases, to hepatitis B vaccination [11]. While some studies have reported LP-like reactions in association with interferon-based treatment for hepatitis B, the data on vaccination-related cases remain limited and largely anecdotal [12]. Specifically, the literature on oral manifestations such as OLP or OLL following hepatitis B immunization is confined to case reports and small case series [7], leaving a knowledge gap regarding the broader epidemiological relevance of such findings. This is particularly relevant given the worldwide prevalence of OLP, which has been estimated at approximately 1–2%, with notable geographic variation [13].

**Table 3:** Analysis of the risk of developing oral lichen planus/oral lichenoid lesion in cohort I (patients who were vaccinated against hepatitis B) and cohort II (individuals who were not vaccinated against hepatitis B)

Risk analysis									
			Cohort		Patients in cohort	Patients with outcome	Risk		
			I	Hepatitis B vaccine	112,444	423	0.376%		
			II	No hepatitis B vaccine	112,444	316	0.281%		
						CI95%		p	
			**Risk difference**		0.1%	(0.001–0.002)		<0.001	
			**Risk ratio**		1.400	(1.210–1.619)			
			**Odds ratio**		1.401	(1.211–1.622)			

This real-world data study was designed to evaluate whether hepatitis B vaccination is associated with an increased risk of OLP/OLL. The results show a statistically significant difference between vaccinated and unvaccinated individuals. However, the observed risk increase was modest (0.376% vs. 0.281%), and the calculated risk and odds ratios (RR and OR ~ 1.4) indicate a relatively small effect size. While this supports the hypothesis of a possible association, it does not establish causality.

Although statistical significance was achieved, the limited clinical relevance of the observed effect size warrants emphasis. An absolute risk increase of 0.1%, with corresponding RR and OR values of approximately 1.4, suggests only a minimal elevation in risk on the population level. This modest difference should be interpreted cautiously, particularly in the context of large-scale public health recommendations.

The underlying mechanisms by which vaccination could contribute to OLP/OLL onset are not fully understood. One theory proposes that heightened cytokine release following immunization may trigger autoimmune processes in predisposed individuals [6, 14]. Another hypothesis suggests molecular mimicry: similarities between viral surface proteins and epithelial antigens might induce CD8 + T-cell-mediated apoptosis in basal keratinocytes [15]. Although these mechanisms are biologically plausible, they remain speculative in the absence of direct evidence. A comprehensive review by Segal et al. (2018) further supports the plausibility of vaccine-induced autoimmunity via molecular mimicry and related mechanisms such as bystander activation and epitope spreading [16].

Several limitations must be considered. First, due to the retrospective nature of the study, residual confounding cannot be excluded. While propensity score matching accounted for known risk factors, variables such as dental restorations, prosthetic materials, and oral hygiene products—known to influence OLL risk—were not available in the dataset [17]. This includes potential effects from corrosion of amalgam fillings or galvanic interactions between dissimilar dental metals, which have been suggested as risk factors in earlier case-control studies [18].

Additionally, psychological factors—which have been hypothesized to influence the development of OLP/OLL—were not documented in the TriNetX database and could therefore not be assessed. Furthermore, detailed geographic information was not available at the level of individual healthcare organizations due to the anonymized nature of the TriNetX platform. However, given that a substantial proportion of participating institutions are known to be located in North America, it is likely that the majority of cases in this study originated from this region. Second, the diagnostic codes used (L43, L44.2, K13.7) encompass both OLP and OLL, which are difficult to distinguish clinically and histopathologically [5]. Although histological confirmation was required, the overlap between entities may still affect interpretation.

To minimize potential bias, the analysis focused on a narrow 30-day follow-up period after vaccination. This reduces the chance of unrelated OLL/OLP onset being misclassified but also limits conclusions about long-term effects. Finally, while the sample size was large and multicenter in origin, the generalizability of findings may be constrained by regional documentation practices and variable data quality across healthcare systems. In summary, this study found a statistically significant but clinically limited association between hepatitis B vaccination and the onset of OLP/OLL. Due to its retrospective design and limited confounder control, the analysis cannot establish causality and should be regarded as hypothesis-generating. Prospective studies are warranted to further explore this association. Future prospective studies with refined diagnostic criteria and more comprehensive confounder control– particularly regarding local oral factors such as dental materials or hygiene-related exposures– are necessary to explore potential causal links.

## Conclusion

In this large-scale real-world data analysis, a statistically significant association was observed between hepatitis B vaccination and the onset of OLP or OLL. However, the absolute risk increase was small, and the effect size was modest. Due to the retrospective and observational nature of the study, causality cannot be established. The findings should therefore be interpreted with caution and regarded as hypothesis-generating rather than confirmatory. Future prospective studies with rigorous diagnostic criteria and comprehensive control of local and systemic confounders are warranted to clarify the potential role of hepatitis B vaccination in the pathogenesis of OLP/OLL.

## Data Availability

The data that support the findings of this study are available from TriNetX. Access to the data is subject to license agreements and privacy regulations and is therefore not publicly available. However, data may be made available from the corresponding author upon reasonable request and with permission from TriNetX.

## Abbreviations

ACE: Angiotensin conversion enzyme
CI: Confidence interval
COVID: Coronavirus disease
HBV: Hepatitis B virus
HCV: Hepatitis B virus
HBsAg: Hepatitis B surface antigen
HCO: Healthcare organization
NSAIDs: Non-steroidal anti-inflammatory drugs
OLL: Oral lichenoid lesions
OLP: Oral lichen planus
OR: Odds ratio
RR: Risk ratio
